# A Text-Book of Animal Physiology

**Published:** 1889-12

**Authors:** 


**A Text-Book of Animal Physiology.** With an introductory chapter on General Biology, and a Full Treatment of Reproduction. For students of human and comparative (veterinary) medicine, and of general biology. By WESLEY MILLS, M. A., M. D., L. R. C. P. (Eng.); Professor of Physiology in McGill University, and the Veterinary College, Montreal. With over 500 illustrations. 8vo, pp. xxii, 700. New York: D. Appleton & Co. 1889.

This work marks a distinct advance in the method of teaching the subject of physiology, and we trust it will be followed by all the progressive teachers in that department. Together with the author, we retain a vivid remembrance of how, during our student days, we were filled with facts and details of technical physiological experiments, until we lost sight almost entirely of the important truths these experiments were intended to illustrate and explain. By the plan of Dr. Mills, the principles of the science of physiology are always kept before the student, and continually reappear in all parts of the book. Technical details are made subordinate to the effort to make clear the laws governing all the phenomena of life. The author's object is finely stated in the opening words of the work:

> The comparative method, the introduction of the teaching of embryology and of the welding principles of evolution, as part of the essential structure of zoölogy, may be said to have completely revolutionized that science; and there is scarcely a text-book treating of that science, however elementary, which has not been moulded in accordance with these guiding lines of thought. So far as I am aware, this cannot be said of a single book on the subject of physiology. Feeling, therefore, that the time had come for the appearance of a work which should attempt to do, in some degree, at least, for physiology what has been so well done for morphology, the present task was undertaken.

How well this attempt has succeeded will be apparent to every one upon an examination of the work.

The task the author set himself was not a simple one, and necessitated, among other things, an entire change in the plan of the book, as compared with all other works on the subject. In the first place, there are no chapters, though the general divisions are headed with larger type, indicating the subject-matter following. Concerning this, Dr. Mills says that observation has taught him that the arrangement into chapters, often gives the student the idea that each function of the body is discharged very much independently; he, therefore, has made a persistent effort throughout the work to impress upon the student the absolute dependence of all parts. In this he has succeeded admirably.

Again, the book has not been overcrowded with elaborate methods of investigation. Enough, however, has been given to show their importance, and to enable each one "to verify the essential truths of physiology," by the more simple and direct methods.

At the end of each subject, a summary is presented, giving in a few concise words what has preceded. This is especially valuable, not only to students, but to all who may consult the work to refresh their physiological knowledge. The subject of Reproduction appears early in the book, instead at the very last, as in most others. The author gives his reasons for this as follows :

> An attempt has been made to use embryological facts to throw light upon the different functions of the body, and especially their relations and interdependence. It, therefore, became necessary to treat this subject early. It is expected, however, that the student will return to it after reading the remaining chapters of this work.

Anothor important feature is the introduction of clinical and pathological facts. This accomplishes two purposes: it serves to teach and impress proper physiology, by showing what the departure therefrom produces; and it illustrates the bearing physiology has upon practical medicine, and is a direct proof of its importance.

Other features to which the author alludes might be mentioned here, but enough has been said to show the general plan of the work. Let us now consider a part of the book in detail—we have not space for a complete analysis—in order to indicate the thoroughness with which Dr. Mills has done his work.

We first have some remarks under the head of General Biology, giving the student some general laws in regard to the nature of all living things. The Cell is then considered, because all living things, whether great or small, are made up of cells. This leads to a description of the simplest forms of life, as illustrated in the unicellular plants, examples of which are the yeast plant and the protococcus. Unicellular animals naturally come next, and we begin to observe a somewhat higher form of life. Examples of these are the amoeba, the parasite organisms, and the bacteria. Animals of a single cell, but with a differentiation in structure, follow, and then we have the multicellular organisms. After which the cell is reconsidered, and its properties discussed, as we have seen them under the previous heads, and some general conclusions are drawn as to the nature of protoplasm, the principal constituent of the cell.

The fact that no two masses of protoplasm are exactly alike, and that there is a physiological division of labor, is shown in the study of the animal body, its construction, and its needs. That one part is functionally dependent upon another is also very beautifully shown. Dr. Mills then presents the difference between living and lifeless matter, taking as his illustration the old comparison between the modern watch and a living organism. We have never seen this more graphically done. After reading it, the student will never forget the fundamental differences existing in matter that is living and matter that is lifeless.

In regard to the classification of the animal kingdom, the author gives that of Claus, but says truly that all classifications are more or less artificial, and, therefore, unsatisfactory. Nevertheless, they serve a useful purpose in helping to simplify knowledge, and cannot be entirely disregarded.

The next divisions are of especial interest. They discuss Man's Place in the Animal Kingdom, and certain general laws governing the manifestations of living matter—such, for instance, as the law of periodicity, or rythm, and the law of habit. We believe that Mr. Herbert Spencer was one of the first to call attention to the law of rythm, and the beauty of the chapter entitled The Rythm of Motion, found in his First Principles, will be recalled by all. We have looked through several works upon Physiology, and can find scarcely a reference to the law. They seem to think it would be out of place in a text-book, for, of course, the authors were not ignorant of it. It is their method, not themselves, that is at fault. So with the laws of habit as well as some others. Dr. Mills deserves the thanks of all students in thus teaching them to know and appreciate the general forces at work, that go to make up the complex phenomena of living things. We trust his effort in this direction will not be in vain.

The next division considers the Origin of the Forms of Life, in which the doctrine of evolution is carefully studied. The argument is arranged under the following heads: Morphology, Embryology, Mimicry, Rudimentary Organs, Geographical Distribution, Paleontology, Fossil and Existing Species, Progression, and Domesticated Animals. The summary of this part says:

> Every group of animals and plants tends to increase in numbers in a geometrical progression, and must, if unchecked, overrun the earth. Every variety of animals and plants imparts to its offspring a general resemblance to itself, but with minute variations from the original. The variations of offspring may be in any direction, and, by accumulation, constitute fixed differences, by which a new group is marked off. In the determination of the variations that persist, the law of the survival of the fittest operates.

This leads directly to the study of Reproduction, which comes next. Its introduction thus early in the work has already been referred to. It occupies seventy-six pages of the book, and is presented in such a way as to attract the student. This is a great gain, for usually it does not receive from them the proper attention.

Then occur divisions with the following titles: Organic Evolution Reconsidered; Chemical Constitution of the Animal Body; Physiological Research and Physiological Reasoning; and we come to the study of the Blood, where most works upon physiology begin.

It is unnecessary to follow the author further. We have seen how radical is the difference between this and the ordinary text-book, and enough has been said also to show its superiority. In a general way, we will say that the rest of the work exhibits the same careful statement, the same comprehensive grasp, the same simple direct way of putting things, and the same beauty of expression as the part already considered. It is a work redounding to the credit of the author, and of great importance to the student.

A word should be said as to the appearance of this book. The publishers seem to have spared nothing to give it a fitting form. The printing, binding, and paper are of the highest order. There are over 500 illustrations of great utility and of fine execution. Some of them are old, familiar friends, but many of them are new and original. Their abundance and their excellence will assist materially in giving a clear understanding of what is now known of physiology. We end with the wish already expressed, that Dr. Mills's work and method will be followed by all progressive teachers, and that all students will be given the benefit of his comprehensive and delightful book. F. H. P.

